# Commercialization report: SpectraWAVE Inc.

**DOI:** 10.1117/1.BIOS.3.3.030501

**Published:** 2026-09-30

**Authors:** Eman Namati

**Affiliations:** SpectraWAVE Inc., Bedford, Massachusetts, United States

## Abstract

This community report discusses the experience of navigating the complex path of commercializing biomedical technologies, offering lessons for the scientific community and industry stakeholders alike.

## 1 Company Overview and Leadership Background

SpectraWAVE was founded in 2017 to develop advanced intravascular imaging technologies for interventional cardiology and coronary artery disease. Its HyperVue system combines optical coherence tomography (OCT) and near-infrared spectroscopy (NIRS) in a single catheter-based platform for use during percutaneous coronary intervention (PCI).

I joined SpectraWAVE in 2020 to help take the company from concept to commercialization. I came to the role with a background in biomedical engineering, medical imaging, and OCT research at Massachusetts General Hospital, as well as executive experience at NinePoint Medical, where I ultimately served as CEO. That startup experience strongly influenced how I approached SpectraWAVE, especially my focus on commercial adoption, manufacturing control, and long-term growth.

## 2 Technology Description

HyperVue (Fig. 1) integrates a next generation proprietary form of OCT, DeepOCT, and NIRS into a single intravascular catheter. DeepOCT provides high-resolution structural imaging of the coronary vessel wall with enhanced depth, while NIRS provides information about plaque composition, particularly lipid burden. Collecting both signals during the same procedure allows physicians to evaluate vessel anatomy and lipid-rich plaque together.

**Fig. 1:**
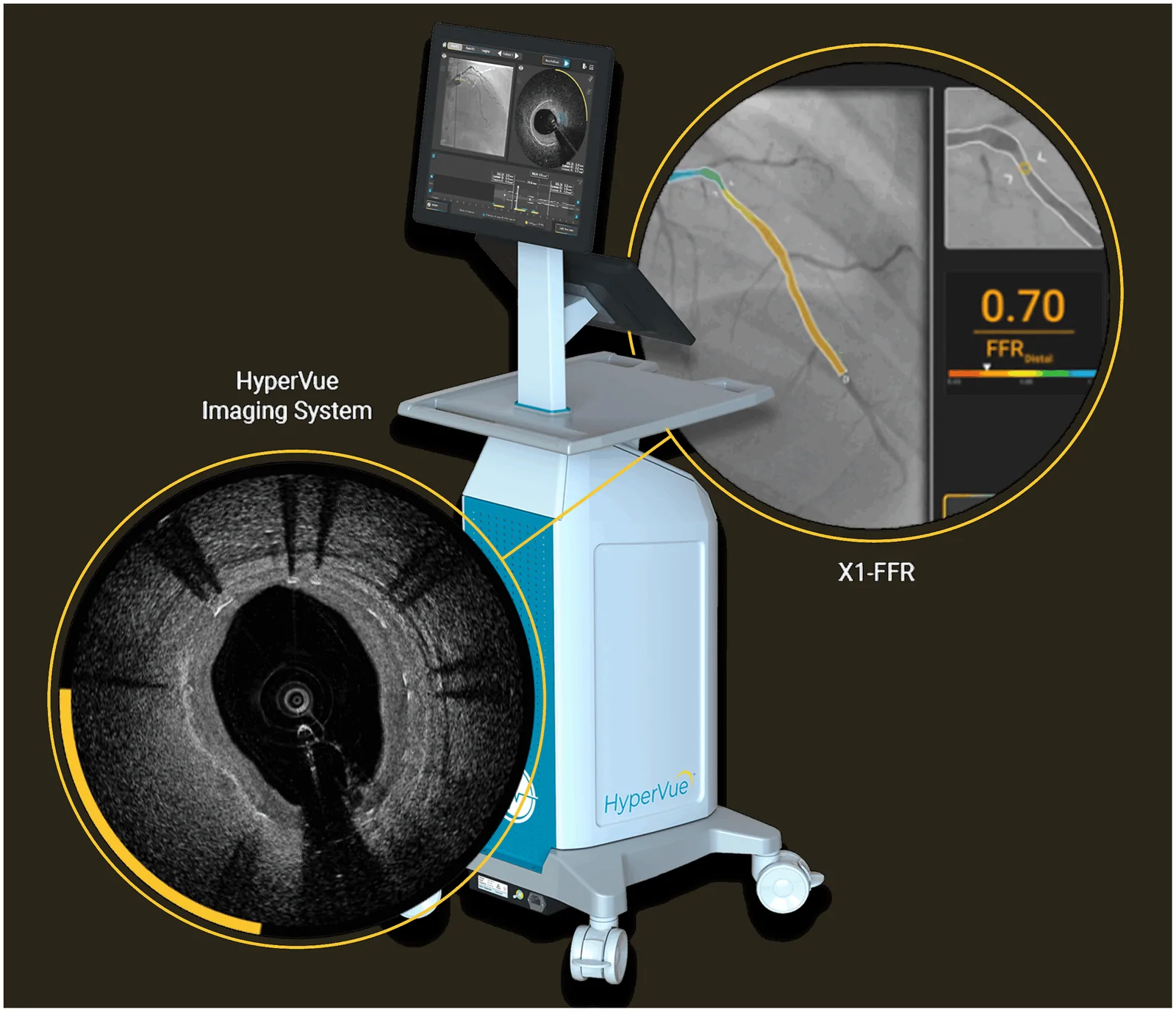
HyperVue imaging system. Image courtesy of SpectraWAVE.

SpectraWAVE also developed AI-enabled software tools that extend the platform beyond imaging. The company has publicly described a broader strategy of using its intravascular imaging hardware as a hub for future AI diagnostic solutions, including tools that support physiologic assessment before stenting.

## 3 Clinical Need and Market

Traditional coronary angiography can show narrowing or blockage in coronary arteries, but it provides limited information about plaque composition and vulnerability. SpectraWAVE was founded on the belief that intravascular imaging would become an increasingly important component of coronary intervention, helping physicians optimize PCI while also identifying high-risk plaques that may contribute to future cardiac events.

That market view was supported by broader changes in interventional cardiology. As professional societies in Europe and the United States strengthened recommendations for intravascular imaging, SpectraWAVE entered a market that was already growing rather than one it had to create on its own.

## 4 Commercialization Stage and Product Development

SpectraWAVE moved from concept to commercial product in under four years. The company built internal teams for design, development, manufacturing, and commercialization, while using external manufacturers for selected subassemblies. Final assembly of both the catheter device and console remained under SpectraWAVE control.

This approach required significant investment, but it gave the company greater control over quality, iteration, and manufacturing readiness. By the time Philips acquired SpectraWAVE, HyperVue had been commercial for approximately ∼18 months and was gaining traction at clinical sites.

## 5 Competitive Landscape

SpectraWAVE competed in an established intravascular imaging market, but differentiated itself by combining DeepOCT, NIRS-based plaque composition assessment, and AI-enabled decision support in a single platform. That combination positioned the company not only as an imaging-device developer, but as part of a broader diagnostic ecosystem for the cath lab.

## 6 Funding History

SpectraWAVE was initially supported by friends-and-family funding and later raised venture capital to support rapid product development, manufacturing, and commercialization. A significant Series B financing included Johnson & Johnson Innovation, through JJDC, Johnson & Johnson’s strategic venture arm.

JJDC was a strategic but relatively neutral investor because its investments do not necessarily lead to acquisition by Johnson & Johnson. That distinction mattered because SpectraWAVE wanted capital and sector credibility while preserving independence and future strategic flexibility.

Philips announced a definitive agreement to acquire SpectraWAVE in December 2025. The acquisition made sense because SpectraWAVE had built a next-generation intravascular imaging product, demonstrated commercial traction, and developed a broader platform strategy that aligned with Philips’ cath lab portfolio.

Several factors contributed to the timing. HyperVue had been commercial for about 18 months, the intravascular imaging market was expanding, and SpectraWAVE had developed AI-enabled physiologic assessment tools that complemented Philips’ existing strengths in intravascular imaging and wire-based physiology.

Philips also recognized that SpectraWAVE had been built for scale. Site visits showed the depth of the company’s investment in product development, manufacturing, and operational infrastructure. Following the acquisition, SpectraWAVE employees remained with the company as part of Philips, preserving the team and culture while creating a path to reach more patients through Philips’ global commercial and operational infrastructure.

## 7 Regulatory Pathway

SpectraWAVE moved through multiple FDA clearances as it developed and expanded its platform. The company received FDA clearance for an AI-enabled physiologic assessment tool in 2025. This tool supports assessment of whether a coronary stenosis should be treated, offering a software-based approach related to fractional flow reserve workflows.

SpectraWAVE’s experience illustrates the importance of regulatory planning for companies building iterative MedTech platforms, particularly when hardware, software, imaging, and AI-enabled decision support are combined.

## 8 Lessons Learned

The first lesson is that impact requires commercialization, not only invention. For technologies intended to improve patient care, academic success and prototype development are not enough. A product must become commercially viable to have broad and sustained clinical impact.

The second lesson is to build for independence, even if acquisition is possible. SpectraWAVE did not assume that a larger company would buy the business. Instead, the company built the team, infrastructure, and manufacturing capacity needed to commercialize on its own. That strategy made the company more resilient and ultimately more attractive to an acquirer.

The third lesson is to choose investors and strategic partners carefully. Corporate venture investment can bring capital, credibility, and strategic insight, but some arrangements may limit future options. SpectraWAVE selected partners in a way that preserved strategic flexibility while supporting rapid growth.

Of course, startup work in MedTech is difficult, with many forces that can slow or derail progress. Money or visibility alone are unlikely to sustain a founder through the process. The stronger motivation is the possibility of building a technology that can improve patient care at scale.

## Supplemental Information

The interview on which this report is based is available in podcast form at Biophotonics Discovery: The Podcast, available wherever you get your podcasts.

